# Effects of low-level prenatal lead exposure on child IQ at 4 and 8 years in a UK birth cohort study

**DOI:** 10.1016/j.neuro.2017.07.003

**Published:** 2017-09

**Authors:** Caroline M. Taylor, Katarzyna Kordas, Jean Golding, Alan M. Emond

**Affiliations:** a Centre for Child and Adolescent Health, School of Social and Community Medicine, University of Bristol, UK; b Epidemiology and Environmental Health, School of Public Health and Health Professions, University at Buffalo, Buffalo, NY, USA

**Keywords:** ALSPAC, Pregnancy, Lead, Heavy metals, IQ, Child, Cognition

## Abstract

**Background:**

The association between childhood exposure to lead (Pb) and deficits in cognitive function is well established. The association with prenatal exposure, however, is not well understood, even though the potential adverse effects are equally important.

**Objectives:**

To evaluate the association between low prenatal exposure to lead and IQ in children, to determine whether there were sex differences in the associations, and to evaluate the moderation effect of prenatal Pb exposure on child IQ.

**Methods:**

Whole blood samples from pregnant women enrolled in ALSPAC (n = 4285) and from offspring at age 30 months (n = 235) were analysed for Pb. Associations between prenatal blood lead concentrations (B-Pb) and child IQ at age 4 and 8 years (WPPSI and WISC-III, respectively) were examined in adjusted regression models.

**Results:**

There was no association of prenatal lead exposure with child IQ at 4 or 8 years old in adjusted regression models, and no moderation of the association between child B-Pb and IQ. However, there was a positive association for IQ at age 8 years in girls with a predicted increase in IQ (points) per 1 μg/dl of: verbal 0.71, performance 0.57, total 0.73. In boys, the coefficients tended to be negative (−0.15, −0.42 and −0.29 points, respectively).

**Conclusion:**

Prenatal lead exposure was not associated with adverse effects on child IQ at age 4 or 8 years in this study. There was, however, some evidence to suggest that boys are more susceptible than girls to prenatal exposure to lead. Further investigation in other cohorts is required.

## Highlights

• The association between prenatal exposure to lead and deficits in offspring cognitive function is not well established.
• Our aim was to evaluate the association between prenatal lead exposure and child IQ at age 4 and 8 years in an observational birth cohort study.
• There was no association of prenatal lead exposure with child IQ at either 4 or 8 years old.
• There was a suggestion, however, that boys are more susceptible than girls to prenatal exposure to lead.

## 1 Introduction

Lead is a toxic metal that is widespread in the environment from natural and anthropogenic sources. Recent years have seen great progress in lead abatement measures particularly in high-income countries, primarily through the removal of lead from petrol and paint, so that the main sources of exposure are now water, dust and soil, food and drink (European Food Safety Authority Panel on Contaminants in the Food Chain, 2010), and cigarette smoke (Taylor et al., 2013). This has resulted in an overall decline in child blood Pb (B-Pb) levels, mirrored by a progressive reduction in the US ‘level of concern’ for B-Pb levels from 60 μg/dl in the 1960s to 10 μg/dl in 1991 and to a ‘reference value’ of 5 μg/dl in 2012 (Centers for Disease Control and Prevention, 2012). Concurrently, however, there has also been a growing realisation that there are effects of lead on child development at all levels of exposure, and that there is no lower limit of safety (Bellinger, 2008, Chandramouli et al., 2009).

Because lead is transferred freely across the placenta (the ratio of fetal:maternal blood lead is about 0.7–0.9 (Rudge et al., 2009, Schell et al., 2003)) and across the blood–brain barrier, in utero exposure to lead may contribute to adverse cognitive outcomes: experimental animal studies suggest that the fetus is particularly vulnerable to the effects of lead because of effects on processes critical to the development of the central nervous system (Basha and Reddy, 2015, Ryzhavskii et al., 2008). Thus, relatively low levels of exposure that do not greatly harm the mother may have a profound effect on the development of the fetus, and on subsequent development and behaviour during childhood. Associations of child IQ with in utero lead exposure are less well established than those for lead exposure during childhood and findings thus far have been inconsistent: several studies have reported adverse associations (Jedrychowski et al., 2009b, Liu et al., 2014b, Wasserman et al., 2000), but others have not found any associations (Cooney et al., 1989, Ris et al., 2004). The timing of the exposure may be critical in relation to developmental time points and consequent adverse effects: Hu et al. (2006) found adverse associations for B-Pb measured in the first trimester but not in later trimesters, but others have found the converse (Kim et al., 2013, Schnaas et al., 2006).

There is also a suggestion from early studies on childhood exposure that there is a differential effect of childhood exposure by sex, with either boys being affected more than girls (Pocock et al., 1987) or vice versa (Tong et al., 2000); analysis of prenatal lead exposure appears to show that boys are more sensitive to exposure than girls, but there are few studies in this area, and thus it is difficult to draw definitive conclusions (Dietrich et al., 1987, Jedrychowski et al., 2009a, Ris et al., 2004).

The aim of the present study was to investigate the association between prenatal exposure to lead and child IQ measured at 4 and 8 years of age in a large sample of mother–child pairs enrolled in a UK birth cohort study (Avon Longitudinal Study of Parents and Children, ALSPAC). A further aim was to investigate the effect of child sex on the possible association. A role for prenatal lead exposure as a primer for the moderation of adverse effects of subsequent exposure in childhood has been suggested (Bellinger et al., 2016): the investigation therefore also included an evaluation of the moderation effect of prenatal B-Pb on the association between child B-Pb and child IQ.

## 2 Methods

### 2.1 The ALSPAC study

The study sample was derived from the ALSPAC, a population-based study investigating environmental and genetic influences on the health, behaviour and development of children. All pregnant women in the former Avon Health Authority with an expected delivery date between 1 April 1991 and 31 December 1992 were eligible for the study; 14,541 pregnant women were initially enrolled, resulting in a cohort of 14,062 live births (Boyd et al., 2013). Further details of ALSPAC are available at www.bris.ac.uk/alspac, where all data in the study can be searched using a data dictionary (http://www.bris.ac.uk/alspac/researchers/data-access/data-dictionary/).

### 2.2 Ethics approval

Ethics approval for the study was obtained from the ALSPAC Ethics and Law Committee and Local Research Ethics Committees.

### 2.3 Exposure measures: collection, storage and analysis of blood samples

Whole blood samples were collected in acid-washed vacutainers (Becton and Dickinson, Oxford, UK) by midwives as early as possible in pregnancy. The median gestational age at the time of blood sampling was 11 weeks (interquartile range 9–13 weeks). Whole blood samples were stored in the original tube at 4 °C at the collection site before being transferred to the central Bristol laboratory within 1–4 days. Samples were at ambient temperature during transfer (up to 3 h). They were then stored at 4 °C until analysis.

Inductively-coupled plasma mass spectrometry in standard mode (R. Jones, Centers for Disease Control and Prevention (CDC), Bethesda, MD, USA; CDC Method 3009.1) was used to measure blood levels with appropriate quality controls (Taylor et al., 2013). The analyses were completed on 4285 women. One sample had a lead level below the limit of detection (0.24 μg/dl). This sample was assigned a value of 0.7 times the lower limit of detection (limit of detection/√2) to reflect the log-normal distribution (Centers for Disease Control and Prevention, 2005, Hornung and Reed, 1990).

A randomly selected sample of parents enrolled in ALSPAC (10%) were invited to bring their children to a research clinic at 30 months of age (Children in Focus) and provided consent for their children to give a venous blood sample. This was analysed for lead by atomic absorption spectrometry as described by Chandramouli et al. (2009) (n = 582).

### 2.4 Outcome measures: IQ

At age 4 years, a subsample of children enrolled in ALSPAC (the Children in Focus cohort), chosen at random from the last 6 months of ALSPAC births (about 10% of the participants), was invited to attend a research clinic for testing. At age 8 years, all children enrolled in the main cohort were invited to attend the clinic. For both clinics, the exact age at testing was recorded.

The outcome measures for this study were verbal IQ, performance IQ and full-scale (total) IQ. All tests were administered by trained psychologists. Inter-rater reliability was ensured by a senior psychologist who observed each tester, met with the testers regularly to discuss the precise administration of the test, and supervised and checked the scoring. Mental development at age 4 years was measured using the Wechsler Pre-school and Primary Scale of Intelligence – Revised UK edition (WPPSI) (Wechsler, 1990). Mental development at age 8 years was measured by the Wechsler Intelligence Scale for Children WISC-III ^UK^ (Wechsler et al., 1992): a short form of the WISC-III measure was employed, where alternate items were used for all subtests, with the exception of the coding subtest which was administered in its full form. Both tests comprise five verbal and five performance subtests. The verbal subtest scores combine to make up the verbal IQ and the performance scores combine to make the performance IQ. The ten subtest scores combine to produce a full-scale (total) IQ score.

### 2.5 Confounders

Data on other potential confounders were collected from the mothers through four postal self-completion questionnaires during pregnancy. The questionnaires are available from the study website (http://www.bristol.ac.uk/alspac/researchers/resources-available/data-details/questionnaires/). Confounders included in the analyses were selected based on those used in previous studies in the literature and those with p < 0.1 in univariate analysis. These were: family adversity index, housing tenure, household crowding, smoking in the first trimester, alcohol consumption in the first trimester, maternal age at index birth, parity, maternal education, length of time the mother had lived in Avon, child sex, child age at testing, weighted life events score and haemoglobin (Hb) level. The family adversity index was derived from responses to questions asked during pregnancy about ten factors, comprising 18 items in total including: (1) age of mother at first pregnancy; (2) housing; (3) mother's and father's low educational attainment; (4) financial difficulties; (5) relationship with partner; (6) family structure; (7) social network; (8) substance abuse; (9) crime; and (10) psychopathology of the mother (anxiety, depression, or suicide attempts) (full details available in Collin et al. (2016)). Each of the 18 items was assigned a value of 1 if an adversity was present and 0 if it was not present (continuous scale, range 0–18). Housing tenure was categorised as mortgaged/owned versus rented/other. Household crowding was derived from the number of persons in household per number of rooms available and categorised into ≤0.5, >0.5–0.75, >0.75–1 and >1. Smoking in the first trimester, alcohol consumption in the first trimester, maternal age at index birth, parity and child age were included as continuous variables (as number of cigarettes per day, number of alcohol units per day, age in years, number, age in months, respectively). Maternal education was categorised as none/CSE/vocational/O level/A level/degree, and the length of time the mother had lived in Avon as all of life/not all of life. The weighted life events score was derived from a life events inventory of 42 items including illness, death of a family member or friend, divorce, problems at work, financial problems, and physical and emotional abuse (Barnett et al., 1983, Brown et al., 1973). Each life event had 5 response categories indicating not only whether or not the event occurred but also to what extent the respondent was affected by it. If a life event had not occurred, it was scored as 0. For each life event recorded as having occurred, mothers gave the event a subjective scoring from ‘not affected at all’ (1) to ‘severely affected’ (4). The events that affected mothers most severely were weighted as described by Dorrington et al. (2014). Data on the haemoglobin (Hb) level collected at booking in the first trimester were retrieved from the obstetric notes.

### 2.6 Statistical analysis

Statistical analysis was done with SPSS version 23 (IBM Corp., Chicago, IL, USA). Datasets were prepared in two ways and analyses completed on each: (1) dataset with inclusion of cases with complete data on exposure, outcomes and confounders; (2) dataset with multiple imputation (datasets at 4 years and at 8 years prepared separately). This second dataset was prepared by imputation of missing data (using the Multiple Imputation function in SPSS) on confounders for cases that had complete data on exposure and outcome at 4 years and at 8 years with 20 imputed datasets each. Analyses from the dataset with complete data are shown in the main tables and corresponding analyses from the other datasets in Taylor et al. (2017).

Values are reported as mean ± SD. Chi-square tests were used to analyse differences in categorical data, and ANOVA was used to compare continuous values by B-Pb values ≤5 or >5 μg/dl. Univariate and multivariable linear regression models were used to examine the association of B-Pb with verbal, performance and total IQ at age 4 and age 8 years. Logistic regression analysis was used to examine the effect of B-Pb ≤5 μg/dl on the likelihood of being in the lowest IQ quartile compared with the highest three quartiles elided. The minimally adjusted model 1 included adjustment for sex and age at testing; model 2 included these two confounders with the addition of maternal variables described in detail earlier (education, smoking, alcohol, age, parity, time lived in Avon); model 3 also included family variables (family adversity index, housing tenure, household crowding, weighted life events score). Birthweight and gestational age were not included, as they are considered to be on the common pathway to IQ. Model 3 was repeated but omitting housing tenure and household crowing to address the possibility that these variables were on the causal pathway. Finally, maternal Hb level at booking was added to model 3 in the main analysis to test the role of iron status in the associations.

The term for sex × prenatal B-Pb was added to model 3 to test for interaction. Sex-specific associations of prenatal B-Pb with child IQ were further evaluated by repeating model 3 after stratification by sex.

Data on child B-Pb were used to investigate prenatal Pb exposure as a moderator of any association between child B-Pb and IQ in model 3 using PROCESS macros in SPSS (www.afhayes.com; complete case analysis).

Regression diagnostics (primarily plots of residuals) were used to check that the models fitted the observed data well, to test the assumptions of regression, and to identify any cases that had undue influence on the model.

## 3 Results

### 3.1 Sample characteristics

The study flow chart is shown in Taylor et al. (2017). From the 4285 mothers who had a B-Pb measurement during pregnancy (4316 live births), 404 children had their IQ measured at age 4 years, and 2217 at age 8 years. Of the 582 children who had B-Pb measured at 30 months, 235 had a mother with a B-Pb measurement, of whom 201 had IQ measured at age 4 years and 172 at age 8 years.

The characteristics of participants included and excluded from the study are shown in Taylor et al. (2017): the mothers of children included in the study at age 4 years were more likely to have a higher educational attainment, to be non-smokers, to be older, to have a mortgage or own their own home, to have a longer gestation and have a child with a greater birthweight compared with those who were excluded. They were also slightly more likely to be white than non-white (p = 0.047) (data not shown). The differences in the characteristics of the two groups was similar at age 8 years, but the mothers of children included in the study were also less likely to have lived their whole life in Avon. There was no difference in the proportion of white vs non-white participants that were included/excluded (p = 0.103; data not shown). Maternal characteristics by prenatal B-Pb ≤ or >5 μg/dl for complete cases are shown in Taylor et al. (2017). The mothers with B-Pb > 5 μg/dl and with complete data at 8 years old were more likely to have higher educational achievement, be a smoker and drink alcohol, be older, have a mortgage or own their own house than mothers whose B-Pb was ≤5 μg/dl. They were also likely to have a shorter gestation and be non-white (p = 0.035; data not shown).

At 4 years, 348 children had data for complete case analysis; at 8 years, 1823 had complete data available. Multiple imputation, therefore, increased the number of cases included at 4 years by about 14% and at 8 years by about 18%, but there was little difference in the results from analyses on any of the datasets (Taylor et al., 2017).

### 3.2 B-Pb levels

**Fig. 1:**
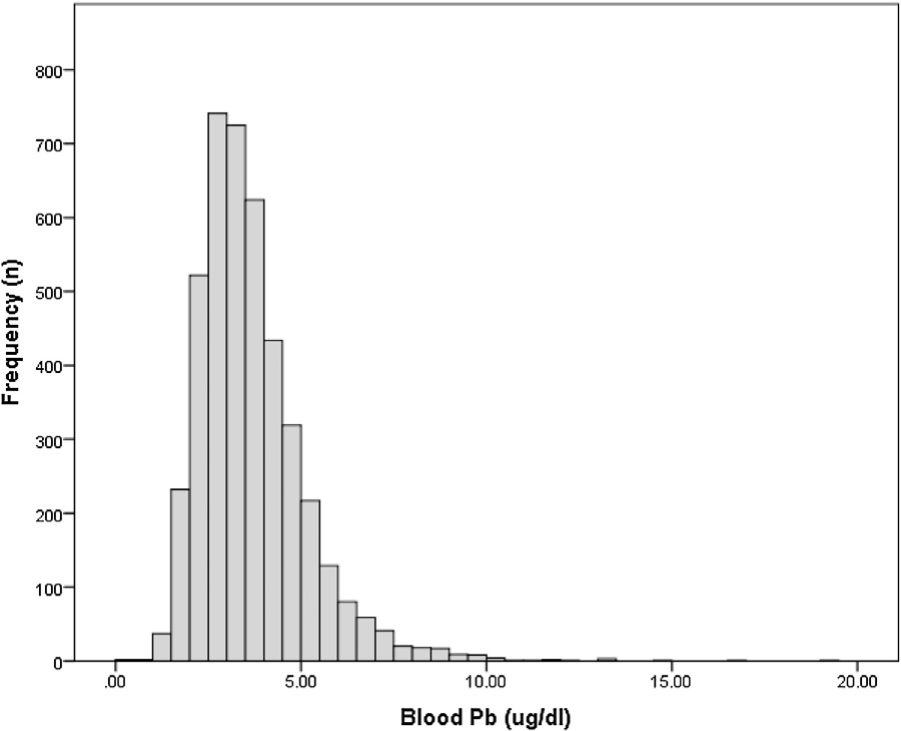
Frequency distribution of B-Pb levels in pregnancy (n = 2451 women who had live births) in ALSPAC.

The mean prenatal B-Pb was 3.67 ± 1.46 (median 3.41, range 0.20–19.14) μg/dl among women who had a live birth (n = 4251). Levels were ≥5 μg/dl (US ‘reference value’; Centers for Disease Control and Prevention (2012)) in 14.3% of women (n = 609) (Fig. 1). The mean child P-Pb was 4.22 ± 3.12 μg/dl (n = 582), with 26.6% having levels ≥5 μg/dl. The correlation between prenatal and child B-Pb was Pearson’s r = 0.280, p< < 0.001.

### 3.3 Associations between B-Pb and IQ

**Fig. 2:**
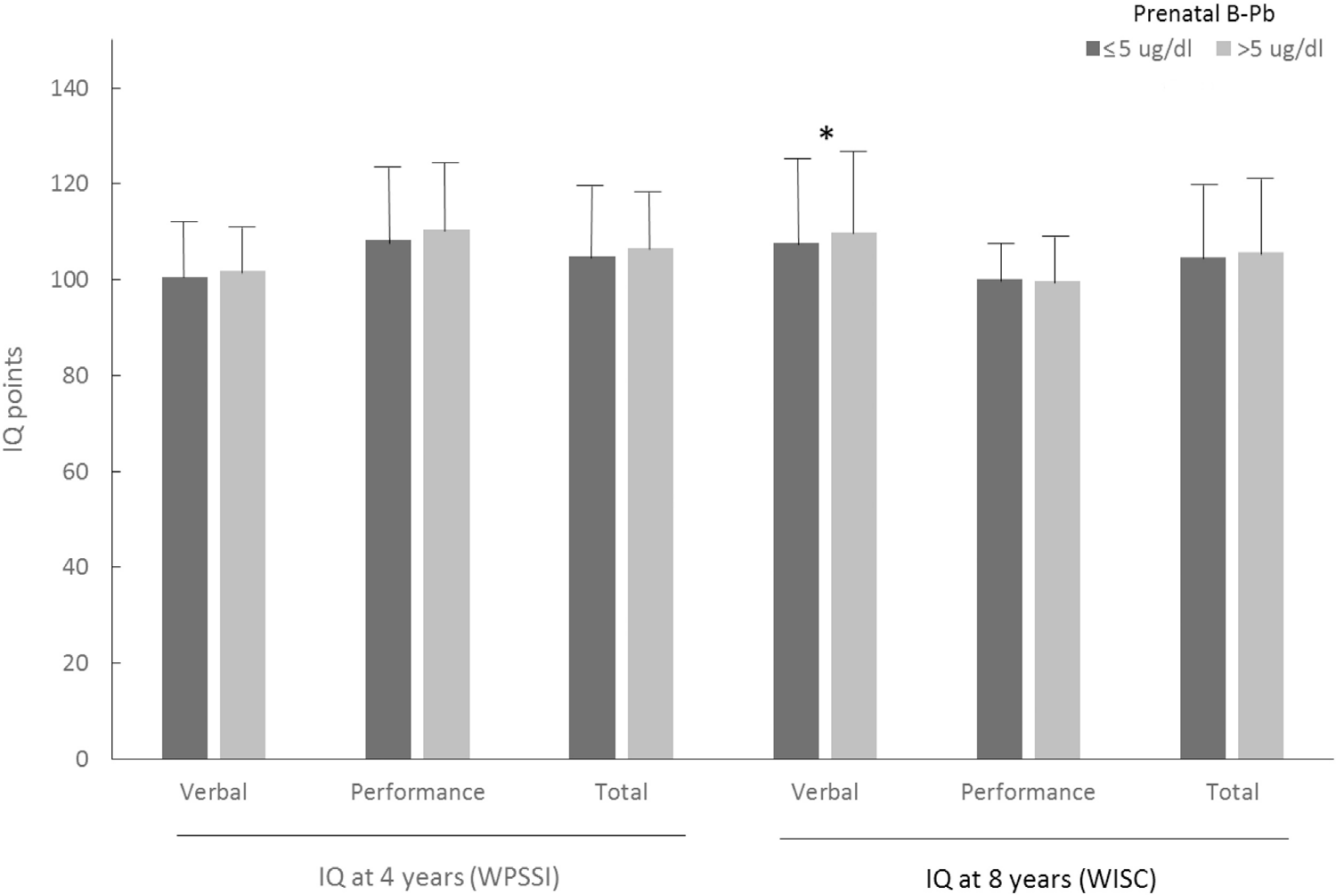
Mean IQ scores by categories of prenatal B-Pb category in ALSPAC: complete cases. *p = 0.050, all other p values > 0.300 (ANOVA). At age 4 years n = 307 for ≤5 μg/dl and n = 41 for >5 μg/dl; at age 8 years n = 1557 (n = 1554 for Total IQ) for ≤5 μg/dl and n = 269 for >5 μg/dl.

There was no evidence for any differences in 4-year IQ scores by prenatal lead category (**≤**5 or >5 μg/dl**)** years in univariate analysis. At age 8 years, verbal IQ was 2.0% greater for prenatal B-Pb >5 than for **≤**5 μg/dl (p = 0.050), but there were no differences for performance or total IQ (Fig. 2).

There was no evidence for any associations of prenatal B-Pb as a continuous variable with IQ at age 4 years in any of the three linear regression models, and this was also the case for performance IQ at age 8 years. For verbal and total IQ at age 8 years, however, there was evidence for positive associations in model 1, but these associations were completely attenuated with further adjustment in models 2 and 3 (Table 1). The inclusion of confounders related to the mother, which included maternal education, in model 3 resulted in the greatest effect size (R^2^) (Table 1). The omission of the variables housing tenure and household crowding made little difference to the output (data not shown). Maternal education was consistently the most influential confounder (explaining more of the variance that other confounders) for each of the three IQ measures at both ages (R^2^ for maternal education in model 3 ranging from 0.018 for verbal IQ at 4 years to 0.047 for total IQ at 8 years; Taylor et al. (2017)). The results of logistic regression with prenatal B-Pb predicting the likelihood of being in the lowest IQ quartile similarly showed no evidence for excess risk at age 4 or 8 years in model 3 (Table 2). Equivalent analyses to those shown in Table 1, Table 2 for all cases and with multiple imputation are shown in Taylor et al. (2017): the results were broadly similar.

**Table 1:** Association of prenatal B-Pb on child IQ at age 4 years and 8 years (linear regression) in ALSPAC: complete cases.

Age (years)	IQ test			R^2^	Unstandardised B coefficient (95% CI)^a^	P values	
						B coefficient	Sex × prenatal B-Pb interaction
4	WPPSI (n = 348)	Verbal IQ	Model 1	0.022	0.42 (−0.54, 1.38)	0.394	
			Model 2	0.151	−0.26 (−1.20, 0.68)	0.591	
			Model 3	0.180	−0.32 (−1.29, 0.66)	0.525	0.957
		Performance IQ	Model 1	0.013	0.38 (−0.67, 1.43)	0.475	
			Model 2	0.126	−0.17 (−1.22, 0.88)	0.752	
			Model 3	0.158	−0.24 (−1.32, 0.83)	0.656	0.491
		Total IQ	Model 1	0.020	0.44 (−0.56, 1.45)	0.387	
			Model 2	0.184	−0.27 (−1.24, 0.71)	0.594	
			Model 3	0.204	−0.32 (−1.32. 0.68)	0.529	0.744

8	WISC (n = 1826)	Verbal IQ	Model 1	0.025	0.76 (0.26, 1.26)	0.003	
			Model 2	0.188	0.32 (0.26, 1.26)	0.188	
			Model 3	0.196	0.32 (−0.16, 0.80)	0.193	0.071
		Performance IQ	Model 1	0.018	0.31 (−0.20, 0.81)	0.235	
			Model 2	0.096	0.08 (−0.42, 0.59)	0.744	
			Model 3	0.107	0.11 (−0.40, 0.62)	0.670	0.065
		Total IQ^b^	Model 1	0.026	0.63 (0.14, 1.12)	0.011	
			Model 2	0.182	0.25 (−0.22, 0.71)	0.299	
			Model 3	0.194	0.26 (−0.21, 0.73)	0.278	0.033

See Methods for details of variables.

Model 1: adjusted for sex, actual age at testing.

Model 2: model 1 plus maternal education, smoking in pregnancy, alcohol in pregnancy, maternal age, parity, time resident in Avon.

Model 3: model 3 plus housing tenure, household crowding, family adversity index, weighted life events score.

a Predicted change in IQ (points) per 1 μg/dl B-Pb.

b n = 1823.

**Table 2:** Association of prenatal B-Pb >5 μg/dl with low child IQ at age 4 and 8 years in ALSPAC (logistic regression): complete cases.

Age (years)	IQ test		n	Regression analyses: Model 3	
				Adjusted OR (95% CI)	P
4	WPPSI	Verbal IQ	348	1.24 (0.49, 3.13)	0.647
		Performance IQ	348	1.20 (0.47, 3.09)	0.706
		Total IQ	348	0.81 (0.29, 2.27)	0.690

8	WISC	Verbal IQ	1826	0.73 (0.51, 1.05)	0.090
		Performance IQ	1826	1.09 (0.78, 1.51)	0.616
		Total IQ	1823	0.86 (0.60, 1.22)	0.390

Reference: highest three quartiles of IQ score elided (vs lowest IQ quartile).

See Methods for details of variables.

Model 3: adjusted for sex, actual age at testing, maternal education, smoking in pregnancy, alcohol in pregnancy, maternal age, parity, time resident in Avon, housing tenure, household crowding, family adversity index, weighted life events score.

### 3.4 Evaluation of maternal Hb in the association between prenatal B-Pb and child IQ

There was no evidence for an effect of early-pregnancy Hb concentration on the association between prenatal B-Pb and child IQ (Taylor et al., 2017).

### 3.5 Sex differences

There was no difference in the prenatal B-Pb between girls and boys (3.73 ± 1.55 versus 3.65 ± 1.47 μg/dl, p = 0.199, respectively). At age 4 years, verbal, performance and total IQ scores were 3.1, 3.1 and 3.5 points greater in girls than boys, respectively (p = 0.043, 0.035, 0.019, respectively); at age 8 years, however, verbal IQ was lower (−0.5 points), performance IQ higher (+2.4 points) and total IQ higher (+0.9 points) in girls than boys (p = 0.553, 0.003 and 0.235, respectively) (Table 3). When the interaction term sex × prenatal B-Pb was added to the model 3 linear regression, the term was not significant in the models at age 4 years, but at age 8 years the interaction term was weakly significant for verbal, performance and total IQ (p = 0.071, 0.065 and 0.033, respectively; Table 1). On stratification by sex for linear regression in model 3, there was evidence of a positive association between prenatal B-Pb and IQ at age 8 in girls (model 3 predictions per 1 μg/dl in prenatal B-Pb: verbal IQ +0.71 points (p = 0.021), performance IQ +0.57 points (p = 0.099), total IQ +0.73 points (p = 0.017); Table 3). In boys, the coefficients tended to be negative (−0.15, −0.42 and −0.29 points per 1 μg/dl, respectively; all p > 0.200) (Table 3). Equivalent analyses for all cases and with multiple imputation are shown in Taylor et al. (2017): the results were broadly similar.

**Table 3:** Association of prenatal B-Pb on child IQ at age 8 years by sex in ALSPAC (linear regression): complete cases.

Age (years)	IQ test		IQ scores			Regression analyses: Model 3^a^					
						Boys			Girls		
			Boys	Girls	p	R^2^	Unstandardised B coefficient (95% CI)^b^	p	R^2^	Unstandardised B coefficient (95% CI) b	p
4	WPPSI	n	197	151			197			151	
		Verbal IQ	99.4 ± 12.9	102.5 ± 13.5	0.043	0.153	−0.26 (−1.66, 1.14)	0.718	0.143	−0.11 (−2.21, 0.61)	0.266
		Performance IQ	107.2 ± 15.1	110.3 ± 13.4	0.035	0.174	−0.74 (−2.41, 0.92)	0.377	0.110	−0.25 (−1.68, 1.17)	0.727
		Total IQ	103.5 ± 13.8	107.0 ± 13.7	0.019	0.177	−0.54 (−2.01, 0.94)	0.474	0.227	−0.65 (−2.07, 0.76)	0.364

8	WISC	n	926^c^	900^d^			926^c^			900^d^	
		Verbal IQ	108.2 ± 17.6	107.7 ± 15.9	0.553	0.186	−0.15 (−0.90, 0.60)	0.702	0.222	0.71 (0.11, 1.32)	0.021
		Performance IQ	98.8 ± 17.3	101.2 ± 16.5	0.003	0.101	−0.42 (−1.19, 0.35)	0.287	0.112	0.57 (−0.11, 1.24)	0.099
		Total IQ	104.4 ± 17.2	105.3 ± 15.6	0.235	0.184	−0.29 (−1.02, 0.44)	0.436	0.219	0.73 (0.13, 1.33)	0.017

See Methods for details of variables.

a Model 3: adjusted for sex, actual age at testing, maternal education, smoking in pregnancy, alcohol in pregnancy, maternal age, parity, time resident in Avon, housing tenure, household crowding, family adversity index, weighted life events score.

b Predicted change in IQ (points) per 1 μg/dl B-Pb.

c n = 924 for boys’ Total IQ.

d n = 899 for girls’ Total IQ.

### 3.6 Prenatal B-Pb as a moderator in of the association between child B-Pb and child IQ

There was no evidence for prenatal B-Pb being a moderator of the association between child B-Pb and IQ at age 4 or 8 years (Table 4), although the coefficients tended to be negative.

**Table 4:** Moderation effect of prenatal B-Pb on the association between child B-Pb and child IQ in ALSPAC.

Age (years)	IQ test		Moderation regression analyses (model 3) for prenatal B-Pb × child B-Pb		
			n	*R*^2^	Unstandardised B coefficient (95% CI)
4	WPPSI	n			
		Verbal IQ	168	0.278	−0.30 (−0.68, 0.08), p = 0.119
		Performance IQ	168	0.167	0.04 (−0.46, 0.54), p = 0.879
		Total IQ	168	0.258	−0.18 (−0.63, 0.28), p = 0.442

8	WISC	n			
		Verbal IQ	144	0.192	−0.08 (−0.26, 0.41), p = 0.758
		Performance IQ	145	0.169	−0.30 (−0.88, 0.29, p = 0.320
		Total IQ	144	0.234	−0.21 (−0.69, 0.28), p = 0.397

See Methods for details of variables.

Moderation analysis using PROCESS macros (www.afhayes.com).

Model 3: adjusted for sex, actual age at testing, maternal education, smoking in pregnancy, alcohol in pregnancy, maternal age, parity, time resident in Avon, housing tenure, household crowding, family adversity index, weighted life events score.

## 4 Discussion

We found no evidence for an adverse association of prenatal B-Pb levels measured in the first trimester with child IQ at either 4 or 8 years of age in adjusted regression models. Maternal education was the strongest variable in attenuation of the crude association. In addition, there was no evidence of moderation by prenatal B-Pb of the association between child B-Pb and IQ. However, there was evidence of a positive association for IQ at age 8 years in girls, as shown by a sex × prenatal B-Pb interaction and by a positive association in adjusted regression models stratified by sex. These models predicted an increase of about +0.7 IQ points per 1 μg/dl increase in prenatal B-Pb in girls. In boys, the coefficients tended to be negative (about −0.3 IQ points per 1 μg/dl increase in prenatal B-Pb).

The evidence that moderate, and even low, postnatal lead exposure is adversely associated with child IQ, resulting in permanent effects into adulthood, is consistent. A re-evaluation by Crump et al. (2013) of data first used in a pooled analysis by Lanphear et al. in 2005 supported Lanphear’s conclusion that postnatal lead exposure ≤7.5 μg/dl is associated with intellectual deficits. Estimation of the loss of IQ per unit increase in postnatal blood Pb level is important because it impacts on an individual’s life course trajectory, as well as having a financial and social cost: WHO estimates that each 1 μg/dl increase in B-Pb results in a decrement of 0.25 IQ points (World Health Organization, 2010), while every US$1 spent on reducing hazards in the USA is estimated to have a cost benefit of US$17–220 (Gould, 2009). However, there has been criticism of the design and analysis of studies on postnatal Pb exposure and child IQ, with a call for greater care in interpretation of data (Kaufman, 2001). Specific criticisms include failure to take uncontrolled variables into account, lack or poor measurement of parental IQ, failure to control for multiple comparisons, and lack of quality control (e.g. trained examiners) in measuring children’s IQ. These criticisms apply equally to studies of prenatal exposure. More recent studies have generally been more rigorous in this regard.

The role of prenatal exposure to lead has been less studied than postnatal exposure, but early gestation represents a critical period in determining the child’s cognitive abilities in later life. Indeed, it is possible that it may have a profound effect than postnatal exposure since prenatal exposure coincides with high rates of cell division and differentiation (Bellinger et al., 2016). Lead crosses the placenta freely, and studies in animals have suggested that the blood–brain barrier is particularly ineffective against lead prenatally (Toews et al., 1977). This is compounded later in pregnancy by an increase in the systemic requirement for calcium, resulting in an increase in bone turnover, and subsequent release of lead stored in bone into the blood. Putative mechanisms for damage to the central nervous system may be triggered directly or indirectly (reviewed by Mason et al. (2014)). Despite this, childhood is an additional period of vulnerability to exposure, with some evidence of similar ‘critical windows’ for specific adverse effects (Brubaker et al., 2010, Hornung et al., 2009) and of effects well into adulthood (Reuben et al., 2017).

Studies with measures of prenatal B-Pb (maternal blood as in the present study, rather than cord blood), however, have had conflicting results: some studies have found adverse associations with IQ in childhood (Wasserman et al., 2000) or adverse associations for blood sampled at specific stages of pregnancy only (Hu et al., 2006, Kim et al., 2013, Liu et al., 2014b, Schnaas et al., 2006) or with specific measures of cognition only (Kim et al., 2013), while others have found no associations at all (Cooney et al., 1989, Moore et al., 1989, Ris et al., 2004). Variations in the timing of blood sampling during gestation, the actual blood Pb levels, the IQ measure used and timing of the IQ test, the number of participants (and thus the power of the study) and the statistical modelling techniques make it difficult to compare studies. For example, Schnaas et al. (2006) measured prenatal B-Pb every 8 weeks from 12 weeks of gestation onwards and child IQ was measured at age 6–10 years by full-scale WISC-R, and found an adverse association only in the third trimester (about 28 weeks). There was no evidence of a lower limit for the effect. The authors controlled for maternal IQ, socioeconomic status and child B-Pb amongst other variables. The geometric mean prenatal B-Pb was relatively high at 8.0 (range 1–33) μg/dl. At a similar prenatal B-Pb of 9.1 μg/dl, however, there were no associations of prenatal B-Pb measured at term with child development measured with the Bayley Scales of Mental Development (BSID) Mental Development Index (MDI) at 6, 12 and 24 months and with the McCarthy subscales at 4 years in adjusted models (Cooney et al., 1989). The results are equally varied at low exposure levels: for example, Wasserman et al. (2000) found inverse associations of prenatal B-Pb measured at mid-pregnancy (1.06 ± 0.32 μg/dl) with IQ measures at 3, 4, 3 and 7 years of age (McCarthy General Cognitive Index (GCI) at 3 and 4 years, WPPSI-R at 5 years and WISC-III at 7 years) in adjusted models. In accordance with our results, Kim et al. (2013) found no association of early prenatal B-Pb (geometric mean 1.36 μg/dl, range 0.26–9.10 μg/dl) with development at 3 and 6 months (BSID MDI and Psychomotor Development Index (PDI)) in adjusted models. As well as reflecting the criticisms of Kaufman (2001) mentioned earlier, this variation may also be due to differences in participant numbers and thus the power to detect difference, the timing of blood sampling during gestation, differences in measures of cognitive abilities and timing of their administration in the child’s life, and differences in the covariates used in adjusted models. Analyses using exposure measures other than maternal whole blood (e.g. plasma, cord blood, bone) have similarly shown a mixture of adverse and null results (Gomaa et al., 2002, Hu et al., 2006, Liu et al., 2014a, Shen et al., 1998). It is notable that a previous study in the ALSPAC cohort found a strong association between prenatal B-Pb and the likelihood of preterm birth (Taylor et al., 2015) and preterm birth is itself associated with poor cognitive outcomes (Kerr-Wilson et al., 2012). However, there was no association between moderate preterm birth and IQ in the ALSPAC cohort (Odd et al., 2012). Thus, preterm birth seems unlikely to act as a mediator. A further factor may be dietary iron intake during pregnancy (Shah-Kulkarni et al., 2016). This suggests that iron status during pregnancy may play an important role in modulating the effects of lead exposure on the fetus. We did not find any evidence for a role for maternal Hb in the association between prenatal B-Pb and child IQ in the present study, although it is possible that interactions are more complex than can be tested here. In addition, maternal haemoglobin, particularly measured in the first trimester, may not be a good reflection of fetal iron status (Paiva et al., 2007).

Sex-related differences in susceptibility to environmental toxins are likely to arise due to the modifying effects of sex hormones and genetic/epigenetic differences (Vahter et al., 2007). In the present study, we found evidence for a positive association of prenatal B-Pb in IQ at age 8 years, whereas the association tended to be negative in boys. The positive association in girls may have arisen through confounding that we were unable to control for, which would also tend to attenuate the association in boys, or perhaps by a differential effect of maternal education. In accord with our results, a study of 457 children born in Poland, found adverse associations of low prenatal lead exposure with BSID MDI scores in 3-year-old boys but not in girls (Jedrychowski et al., 2009a), confirming earlier findings of increased susceptibility in boys (Dietrich et al., 1987, Ris et al., 2004). As well as its roles in female reproduction, it is now recognised that oestrogen has a central role in neurodevelopmental processes in both males and females, and that there are differences in the distribution and density of oestrogen receptors in male and female brains (Gillies and McArthur, 2010). Since lead is known to have anti-oestrogenic effects (Kavlock et al., 1996), it is possible that these differences could account for an increased susceptibility in males, with oestrogen having a protective effect in females.

There are several strengths of this study. (1) The study involved large numbers of pregnant women with both prenatal B-Pb and child IQ measured at 8 years. (2) The prenatal exposure was measured in the first half of pregnancy, in contrast to studies which have relied on cord blood Pb or Pb in other matrices such as hair or urine. (3) IQ measures used in the study were well validated and conducted with supervision by trained examiners. (4) IQ at 4 and 8 years was measured by comparable methods, with WPPSI developed for children too young for the full WISC. There are also several limitations. (1) There was a relatively small number of children with IQ measured at age 4 years and relatively low numbers with measures at both ages. The sample numbers in the moderation analysis were particularly small. (2) Even under the supervision of trained examiners, IQ tests are subject to error, based for example, on the child’s boredom, mood, tiredness, and rapport with the examiner. Errors that apply to both the verbal and performance IQ will be compounded in the total IQ. (3) Although we were able to account for many possible confounders in our analyses, there are likely to be others that were unable to adjust for, for example maternal and paternal IQ. This would contribute to any findings being due to chance. (4) The role of concomitant exposure to other pollutants such as cadmium, which might have the effect of masking associations was not included and should be explored further in other studies. (5) The time lapse between the exposure and the outcomes in this study means that the child will have experienced unknown further exposure to lead during childhood, which cannot be accounted for.

## 5 Conclusion

Although the association between child lead exposure and IQ is well established, the evidence for an association between prenatal Pb exposure and child IQ is conflicting. We found no evidence for an association of moderate prenatal B-Pb levels measured in the first trimester with child IQ at either 4 or 8 years of age in adjusted regression models. The timing of the exposure during pregnancy may be critical in determining adverse neurological events, and it is possible that measures of exposure in the second and/or third trimester would have given different results. B-Pb levels were greater in early childhood than during pregnancy in this group and it possible that exposure during childhood has a greater association with adverse cognitive effects than prenatal exposures.

## Competing interests

The authors declare they have no conflicts of interest.
